# Dissociating neural markers of stimulus memorability and subjective recognition during episodic retrieval

**DOI:** 10.1038/s41598-018-26467-5

**Published:** 2018-06-06

**Authors:** Wilma A. Bainbridge, Jesse Rissman

**Affiliations:** 1 Department of Brain and Cognitive Sciences, Massachusetts Institute of Technology, Cambridge, MA USA; 2 Department of Psychology, University of California, Los Angeles, CA USA; 3 Department of Psychiatry and Biobehavioral Sciences, University of California, Los Angeles, CA USA; 4 Laboratory of Brain and Cognition, National Institute of Mental Health, Bethesda, MD USA

## Abstract

While much of memory research takes an observer-centric focus looking at participant performance, recent work has pinpointed important item-centric effects on memory, or how intrinsically memorable a given stimulus is. However, little is known about the neural correlates of memorability during memory retrieval, or how such correlates relate to subjective memory behavior. Here, stimuli and blood-oxygen-level dependent data from a prior functional magnetic resonance imaging (fMRI) study were reanalyzed using a memorability-based framework. In that study, sixteen participants studied 200 novel face images and were scanned while making recognition memory judgments on those faces, interspersed with 200 unstudied faces. In the current investigation, memorability scores for those stimuli were obtained through an online crowd-sourced (N = 740) continuous recognition test that measured each image’s corrected recognition rate. Representational similarity analyses were conducted across the brain to identify regions wherein neural pattern similarity tracked item-specific effects (stimulus memorability) versus observer-specific effects (individual memory performance). We find two non-overlapping sets of regions, with memorability-related information predominantly represented within ventral and medial temporal regions and memory retrieval outcome-related information within fronto-parietal regions. These memorability-based effects persist regardless of image history, implying that coding of stimulus memorability may be a continuous and automatic perceptual process.

## Introduction

Why do we often remember events that are not particularly important to us, yet we forget the faces of acquaintances we desperately try to remember? Much of memory research focuses on answering this question through an *observer-centric* focus, exploring the steps the brain undergoes when forming a memory and trying to explain the factors that lead some subjects to perform better than others. However, an equally important factor is *item-centric* effects – how do stimulus-driven factors influence these steps in making a memory?

In fact, stimulus-driven factors have been found to determine the ultimate mnemonic fate of a given face as much as all other factors combined, including individual differences, environment, and noise^1^. Stimulus-driven factors are intrinsic properties of a stimulus that influence observer behavior. For example, stimuli that trigger bottom-up attention orienting (e.g., threatening faces^2–4^, emotional faces^5,6^ or even just brighter stimuli^7,8^ may tend to cause biases in tasks such as spatial cueing and visual search^9,10^. Another example is with statistical learning, where the relative frequencies and ordering of stimuli affect learned representations of those stimuli^11,12^. One recently characterized stimulus property of relevance to memory in particular is *memorability* – an intrinsic stimulus property reflecting the probability of a given stimulus to be successfully encoded into memory. Stimulus memorability is highly consistent and reliable across observers^13–21^; that is, people tend to remember and forget the same scenes^13^ and faces^1^ as each other, in spite of the diversity in perceptual experience across people. Memorability has been found to be its own stimulus property; for faces, it cannot be modeled as a composite measure of other face attributes such as attractiveness, trustworthiness, or subjective ratings of distinctiveness or familiarity^1^. This consistency of memorability has been found to persist within a face identity across emotional and viewpoint changes in the face, unlike other face attributes^14^; if a person is memorable when they are smiling, then they are also likely to be memorable when facing away. Memorability is thus intrinsic not only to images, but also to dynamic entities such as a person’s identity. Using computer vision metrics, memorability can thus be quantified and even manipulated for a face image^15^. Memorability has also been found to be resilient to different time lags^16,17^, stimulus contexts^18^, and experimental paradigms^19,20^, showing that memorability as a stimulus property generalizes to alternate contexts. And, memorability effects have been shown to be independent from bottom-up attention, top-down attention, and perceptual priming^21^, indicating that memorability is not a mere proxy for other cognitive processes known to be related to memory.

If stimulus-driven memorability is known to play such a strong role in memory behavior, to what degree are previously identified neural effects of memory partially explained by stimulus memorability? Previous work has found strong, stereotyped patterns of neural activity based on stimulus memorability during incidental encoding of an image^22^; however, no work has examined memorability during active memory retrieval. As memorability is easily measurable for a given set of stimuli, previous memory studies could be re-examined with a framework based on stimulus memorability. Several functional magnetic resonance imaging (fMRI) studies have been able to successfully classify brain activation patterns based on individual memory behavior at the stage of retrieval^23,24^. To what degree are these successful classifications influenced by the memorability of the stimuli, or might there even be separate signatures for stimulus-driven memorability versus observer-driven memory behavior?

In this paper, we examine the interaction of stimulus-driven memorability and memory retrieval through a reanalysis of the face stimuli and blood-oxygen-level dependent (BOLD) data from Rissman *et al*.^23^, one of the first fMRI studies to decode memory recognition behavior from brain activity patterns. We find that we are able to significantly classify whether individual face stimuli have high or low memorability based on the BOLD data, and the regions supporting this classification significantly differ from regions able to classify memory behavior. We find these memorability-related patterns remain relatively consistent across both trials where participants successfully recognized old images (hits) and successfully rejected new images (correct rejections). Additionally, we find that support vector regressions trained on activity patterns from these same regions are able to predict the memorability score of individual faces, providing the first evidence for a continuous representation of memorability in the brain. Taken together, these results provide support for memorability as a measurable stimulus property that can be used to analyze neural correlates of memory, and provide new insights into the information that may be relevant to the brain when retrieving an item from memory.

## Results

### Characterizing the Range and Consistency of Memorability

Our first step was to quantify the memorability of the face stimuli from Rissman *et al*.^23^ and see whether these memorability scores had sufficient variance and consistency to be taken as an intrinsic property of each image. In the original experiment^23^, participants (N = 16) studied 200 face images outside of an MRI scanner and then were tested for memory retrieval approximately 1 h later during fMRI scanning with the same 200 images and an additional 200 foil face images. For the current experiment, memorability scores were obtained for these 400 face stimuli using a large-scale Amazon Mechanical Turk (AMT) memory test. 740 AMT workers viewed a pseudorandom sequence of the face images presented for 1 s at a time and pressed a button whenever they saw a repeated face image (see Methods). For each stimulus, we used the group-level AMT data to calculate a hit rate (*HR*; percentage of participants successfully identifying a repeat) and a false alarm rate (*FA*; percentage of participants falsely identifying the first presentation as a repeat). While previous memorability work has focused on *HR* while selecting for low *FA* across stimulus conditions^22^, here we have stimuli that vary in both *HR* and *FA*. For example, while an image with a high *HR* and low *FA* may be truly memorable, an image with a high *HR* and also high *FA* may be one that causes a sense of familiarity. To account for the effects of *FA*, we define memorability henceforth as the corrected recognition score *Pr*, where *Pr* = *HR* − *FA*^25^. Note that *d-prime* was not used here due to the fact *HR* and *FA* were not necessarily derived from the same set of participants.

**Figure 1:**
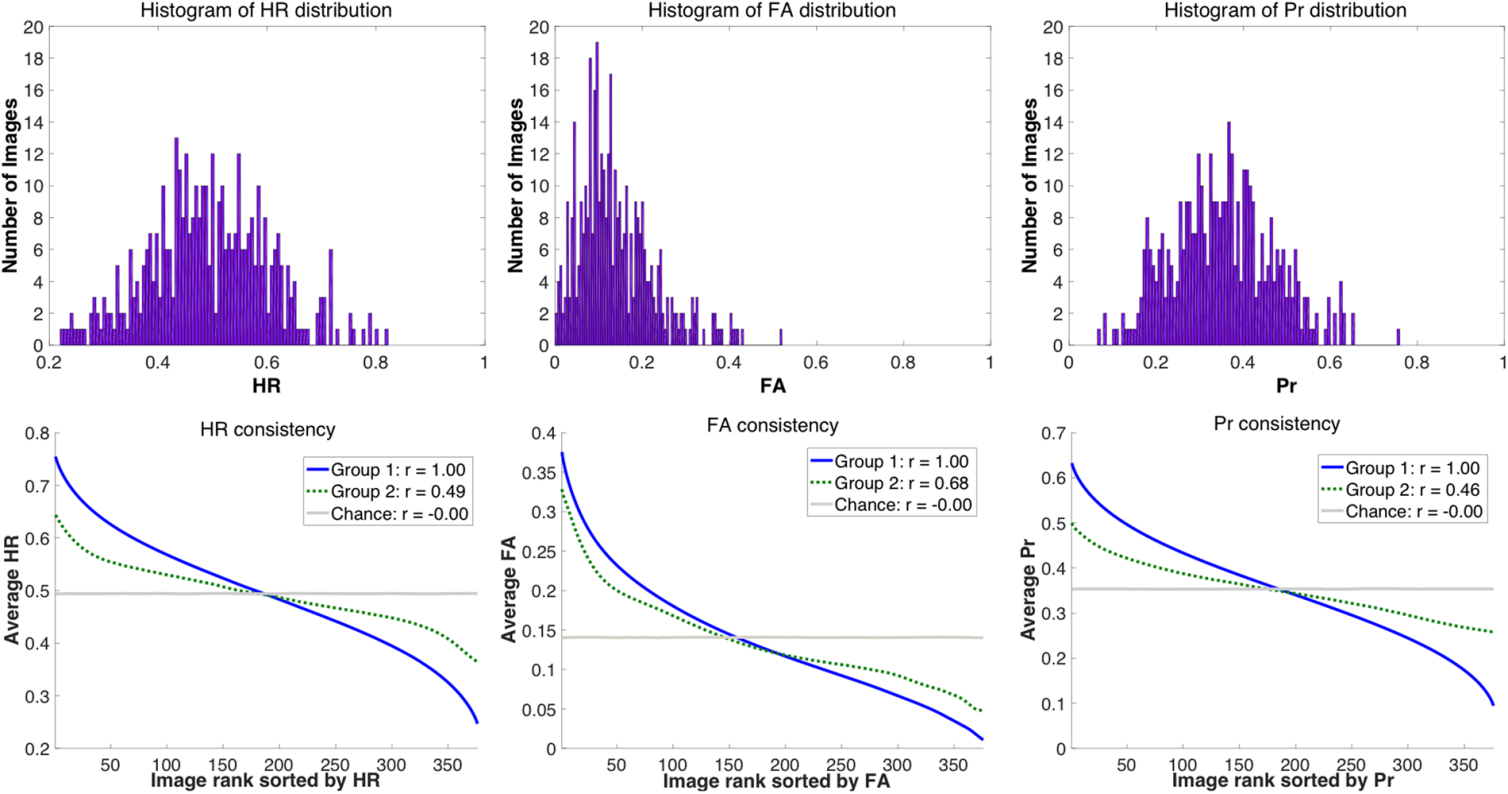
(Top) Histogram distributions of hit rate (*HR*), false alarm rate (*FA*), and memorability score corrected recognition (*Pr*) for an online memory test with the 400 face stimuli. These histograms show a wide range of memorability scores, making them an ideal set for probing the extent of memorability effects in the brain. (Bottom). Split-half consistency analyses (Spearman’s correlations of randomly split halves of the participants, over 10,000 iterations) for *HR*, *FA*, and *Pr*. The blue line indicates the average memorability scores (sorted from highest to lowest) for the first random participant half, while the green dotted line shows the average memorability scores for the same ordering from the second half. The grey line shows an estimate of chance, where scores for the second half are instead shuffled. The r-values indicate average correlation. Overall, these graphs show that participants generally remember (as well as false alarm to) the same images as each other (all *p* < 0.0001).

These stimuli showed a wide range in memorability score *Pr* (*M* = 0.35, *SD* = 0.12, *min* = 0.06, *max* = 0.76). *HR* showed similarly wide range (*M* = 0.49, *SD* = 0.11, *min* = 0.22, *max* = 0.82) as well as *FA* (*M* = 0.14, *SD* = 0.09, *min* = 0, *max* = 0.52). As a point of comparison, *d-prime* also showed a wide range (*M* = 1.15, *SD* = 0.45, *min* = 0.23, *max* = 2.67). A consistency analysis correlating the stimulus memorability rankings of random split halves of participants (see Methods) found significantly high consistency in *Pr* across images (*r*(398) = 0.46, *p* < 0.0001). *HR* was also significantly consistent across observers (*r*(398) = 0.49, *p* < 0.0001) as well as *FA* (*r*(398) = 0.67, *p* < 0.0001) and *d-prime* (*r*(398) = 0.53, *p* < 0.0001); see Fig. 1. This means that while there was a wide range in memory performance across images, observers tended to remember and forget the same images as each other, and so memorability is consistent within an image. These face stimuli are thus an ideal testbed to look at the relationship of various levels of memorability to BOLD data as well as individual memory performance.

**Figure 2:**
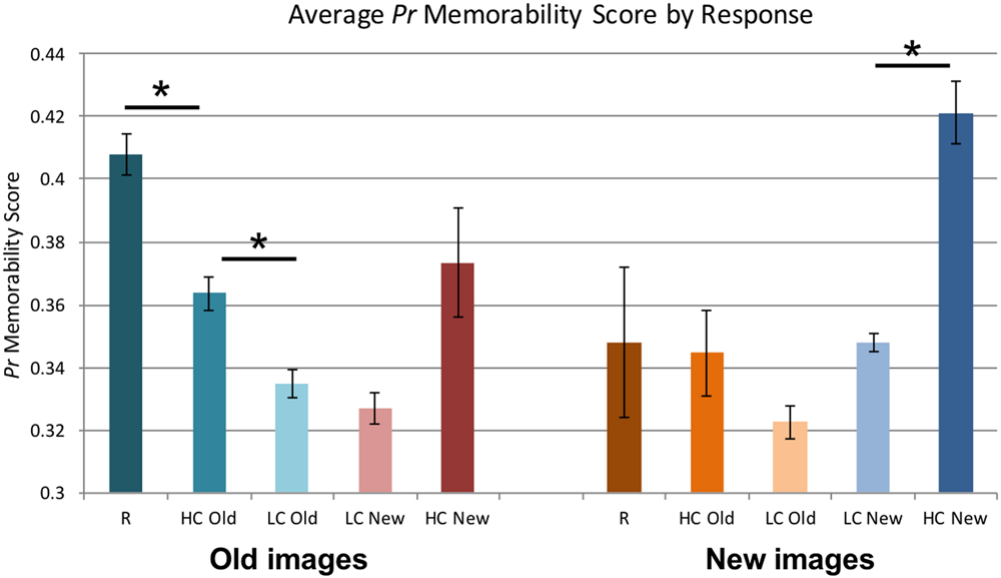
Average *Pr* memorability score (from the online memory test) for each image type (old or new) and response type of the participants in the scanner experiment. Error bars indicate standard error of the mean. Asterisks (*) indicate a significant Bonferroni-corrected pairwise difference between conditions (p < 0.001). All correct trials (e.g., responding “old” for an old image or “new” for a new image) showed significantly higher memorability for images with more confident responses (R = Recollect, HC = high confidence, LC = low confidence). Note that large error bars are seen for trial types with small numbers of trials; all correct response types had on average over 35 trials per participant, while incorrect response types had fewer (e.g., HC New for old images had on average only 11 trials per participant, R for new images had only 2 per participant, and HC Old for new images had only 11 per participant).

The scanned participants’ memory ratings during the retrieval task also highly corresponded to these memorability scores collected online (Fig. 2). While retrieving memories in the scanner, participants had five response options to indicate their memory and confidence: (1) recollected the image (i.e., they could think back to the actual experience of first encountering that photograph), (2) high confidence the image was old, (3) low confidence the image was old, (4) low confidence the image was new, or (5) high confidence the image was new. As expected, for old images, recollected images had the highest memorability scores, with high confidence hits having lower memorability scores, and low confidence hits having even lower memorability scores. A repeated measures within-subjects 1-way ANOVA for participant response for old trials was conducted to test this pattern, excluding the “high confidence new” response for old trials since only five participants had at least five trials of that type. There was a significant main effect of confidence rating on memorability for old images (*F*(3, 45) = 47.11, *p* = 6.22 × 10^−14^, effect size *η*^2^ = 0.759; Mauchly’s test indicates an assumption of sphericity is preserved: *χ*^2^(5) = 3.61, *p* = 0.608), where Bonferroni-corrected post-hoc pairwise comparisons show significantly higher memorability for recollected than high confidence old trials (*p* = 1.46 × 10^−4^), and high confidence old trials than low confidence old trials (*p* = 7.00 × 10^−4^). Likewise, new images that were judged to be new (i.e., correct rejections) with high confidence were also those that tended to get the highest memorability scores in the online sample. A repeated measures within-subjects 1-way ANOVA for participant response for new trials was conducted, excluding the “recollection” response for new trials, since only one participant had at least five trials of that type. There was a significant main effect of confidence rating on memorability for new images (the assumption of sphericity was violated *χ*^2^(5) = 16.94, *p* = 0.005, so the ANOVA was Greenhouse-Geisser corrected (ε = 0.633): *F*(1.90, 26.57) = 18.40, *p* = 1.30 × 10^−5^, effect size *η*^2^ = 0.568). A Bonferroni-corrected post-hoc pairwise comparison showed significantly higher memorability for high confidence new trials than low confidence new trials (*p* = 3.00 × 10^−6^). The patterns for trials where participants’ responses were incorrect (e.g., rating new images as old or vice versa) are less clear, as there were fewer trials of this type (e.g., only 2 trials on average per participant rating a new image as being “recollected”). Overall, these results indicate that memorability scores determined in a separate large-scale online experiment can be successfully applied to account for behavioral performance in an independent set of participants (from the smaller-scale fMRI experiment), even when the images were studied under a very different paradigm and setting.

### Representational Geometries of Stimulus Memorability and Subject Memory across the Brain

**Figure 3:**
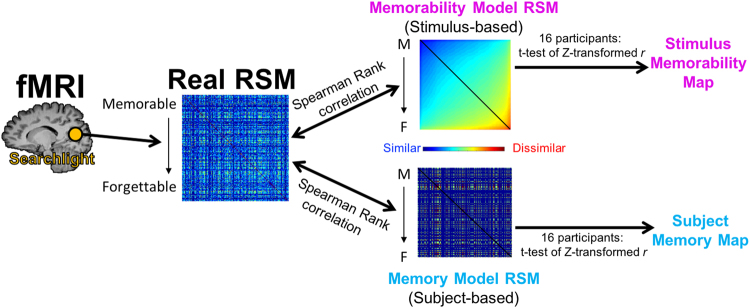
A diagram of how the Representational Similarity Analysis (RSA) was conducted in this study, using toy data. Essentially, voxel data were extracted from searchlights across the brain (real representational similarity matrix, RSM) and then correlated with a RSM based on stimulus memorability (i.e., more memorable stimuli are more similar) and a RSM based on individual subject memory performance (i.e., stimuli an individual remembered are more similar). Stimuli in the matrices are ordered from most memorable (M) to most forgettable (F). Note that the Memory Model RSM only has three colors to represent the three possible values in the matrix (blue = both stimuli were remembered; green = one was remembered and one was forgotten; red = both were forgotten). In contrast, the Memorability Model RSM shows a continuous representation of memorability. The diagonals (colored black) in the RSMs were not used in the analyses, as they will always be 1 (i.e., the similarity of a stimulus to itself). From the correlations of the Model RSMs to the Real RSM, we can form maps of which regions in the brain showed significant correlations across the sixteen participants to the stimulus-based memorability model, and which showed significant correlations to the subject-based memory model.

Since the face stimuli show strong consistency and variance in their memorability, we can then explore their neural correlates during the scanned retrieval task. Specifically, representational similarity analyses (RSAs) allow one to examine the representational geometry of information in the brain^26^, by looking at where hypothesized models of stimulus similarity correlate with neural data. For example, are there regions in the brain that show more similar neural patterns for a pair of memorable images than a pair of forgettable images? Also, while this hypothesis is based on *stimulus memorability*, might there also be regions with a representational geometry based on an individual subject’s *memory* behavior (e.g., where there are more similar neural patterns for two remembered images than two forgotten images), and how might these two sets of regions compare? RSAs were conducted in a whole-brain searchlight using a model based on stimulus memorability and a model based on participant memory behavior and then compared (see Fig. 3 and Methods for further details).

**Figure 4:**
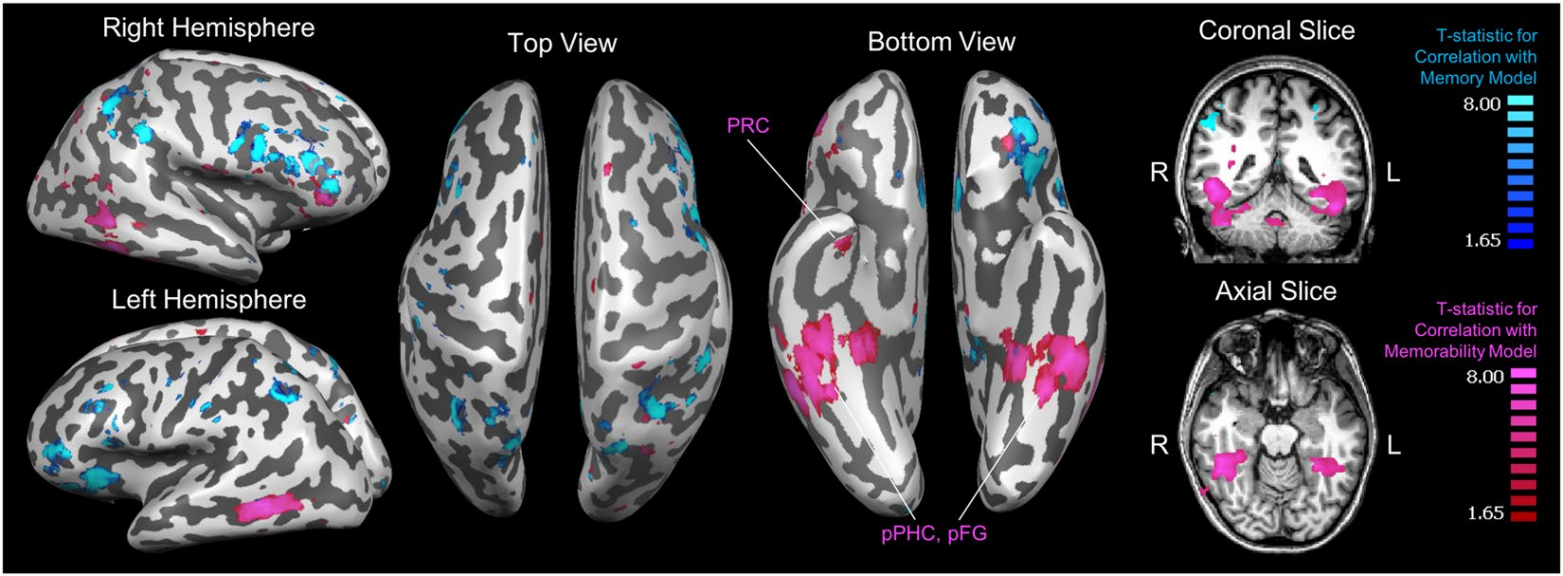
Group *t*-maps of the top 1000 voxels whose local activity patterns (i.e., 7-voxel diameter spherical searchlight) correlated with the stimulus memorability-based representational similarity matrix (RSM, red/pink) and the top 1000 voxels whose local activity patterns correlated with the subject memory-based RSM (blue), shown on a Talairach-space example brain. The two maps have very little overlap (9 voxels, or 0.9%); the memorability-based voxels span the ventral visual stream, the posterior temporal lobe (pPHC = posterior parahippocampal cortex, pFG = posterior fusiform gyrus), and anterior medial temporal lobe (PRC = perirhinal cortex). In contrast, the memory-based voxels span frontal and parietal regions. The lowest cluster-threshold corrected significance level that all 1000 voxels pass for both maps is *p* < 0.001.

We find a dissociation of two strikingly different sets of regions that emerge as being maximally significant for the memorability-based model and the memory-based model (Fig. 4). Specifically, the memorability-based model shows its highest significant correlations with BOLD activity patterns in the ventral visual stream and medial temporal lobe (MTL), overlapping with regions such as the parahippocampal cortex (PHC), fusiform gyrus (FG), and perirhinal cortex (PRC). In contrast, the memory-based model shows its highest significant correlations in frontal and parietal regions. Of the top 1000 voxels of each contrast, only 9 voxels (or 0.9%) are shared between the two contrasts (Refer to Supplementary Figure 1 for a chart of how the amount of voxel overlap varies by number of voxels included in each map). These results indicate that different sets of regions preferentially support representations of stimulus-driven memorability versus the actual memory fate of that stimulus.

### Memorability Across Image Types

While different neural systems may be active for quickly calculating the memorability of an image versus retrieving a specific memory, might memorability-related activity be modulated by the history of the image? That is, will we see different patterns for old images that are successfully remembered versus new images that are correctly rejected? For the former trial type, participants are retrieving a specific item from memory successfully, while for the latter trial type, participants are viewing a novel item and identifying it as being novel. Recognition and novelty detection are different memory behaviors, shown to depend largely upon separate neural substrates^27–29^, and so might there also be similar differences in how the brain processes the memorability of an old stimulus versus a new one?

**Figure 5:**
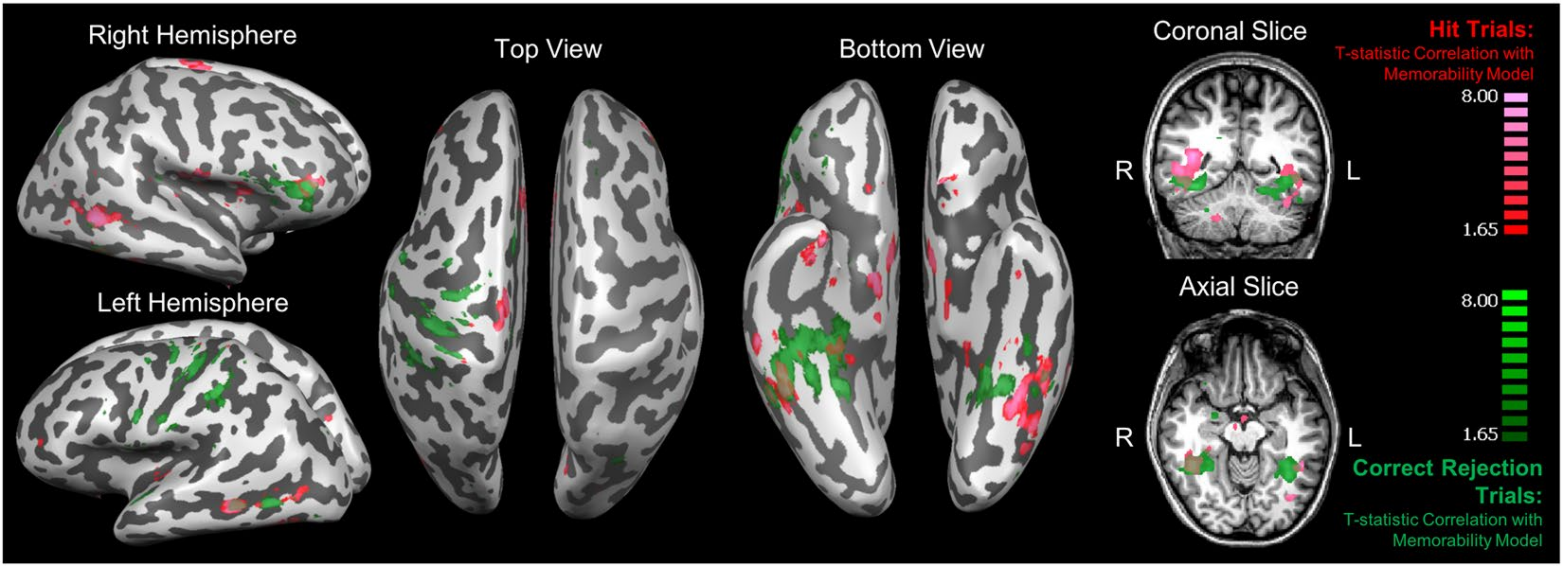
A comparison of the top 1000 significant voxels whose local activity patterns correlated with the memorability-based representational similarity matrix (RSM) constructed only with hit trials (red/pink) and only with correct rejection trials (green). The lowest cluster-threshold corrected significance level that all 1000 voxels pass for both maps is *p* < 0.005. Both maps show significant regions in the bilateral ventral stream and posterior temporal lobe, as well as some regions in the frontal and parietal cortices.

We explored this question using RSA searchlights of memorability for only hit trials, and then also only for correct rejection trials. The number of trials used was downsampled (as described in the Methods) in order to ensure that trial counts were balanced across trial types for each participant. From these searchlights, similar regions for memorability emerge for both hit and correct rejection trials, particularly within the ventral visual stream and posterior temporal lobe (Fig. 5), as seen in the regions that emerge for general memorability (Fig. 4). An analysis of voxel overlap (Supplementary Figure 2) revealed that the maps for hit and correct rejection trials had 93 overlapping voxels (9.3%) in the top 1000 voxels. Hit trials additionally show significant correlations in the anterior temporal pole (encompassing regions within the MTL), while correct rejection trials show correlations in the parietal lobe. Both trial types also show significant correlations with regions in the ventrolateral prefrontal cortex. These maps indicate that hit and correct rejection trials largely show the same regional patterns of memorability (although not necessarily overlapping in the precise anatomical coordinates), indicating that memorability effects during retrieval exist regardless of individual memory behavior. However, there may be subtle differences in the regions that use memorability-based information depending on the specific retrieval process.

### Representational Geometries within Regions of Interest

Representational similarity analyses were conducted within perceptual and memory-related regions of interest (ROIs) in order to examine more specifically which regions are sensitive to measures of memorability, and to probe the differences between hit and correct rejection trials. These ROIs were defined by probabilistic maps made from over 40 participants for MTL regions and 70 participants for ventral visual stream regions^22^, with ROIs taken as the voxels shared by at least 25% of participants (as in^30^). For the MTL, we investigated representational similarity within the perirhinal cortex, entorhinal cortex (ERC), amygdala, hippocampus, and parahippocampal cortex. For the ventral visual stream, we looked at early visual cortex (EVC), and higher-level commonly category-specific perceptual regions of the fusiform face area (FFA), parahippocampal place area (PPA), and lateral occipital complex (LOC).

We find that several perceptual ventral visual stream regions are significantly correlated with a memorability-based RSM (corrected for multiple comparisons using False Discovery Rate FDR, corrected *α* = 0.007). Specifically, significant correlations emerge in the right PPA (*r*(14) = 0.015, *p* = 0.007), the right LOC (*r*(14) = 0.022, *p* = 0.006), and marginal significance for the left FFA (*r*(14) = 0.006, *p* = 0.030; does not survive FDR correction). No significant correlation is found in the EVC (*p* > 0.08). In contrast, no perceptual regions show a significant correlation with the memory-based RSM (all *p* > 0.10). These results mirror the results in the whole-brain searchlight analysis, where memorability is correlated with ventral neural signals, while memory is not. These results implicate memorability as being a high-level perceptual signal, as higher-level perceptual regions (FFA, PPA, LOC) show a correlation with memorability. It is also interesting to note that while only face images were viewed, non-face selective regions (PPA, LOC) show a significant correlation with memorability signals, indicating that sensitivity to memorability may be content-generic across the brain.

In terms of memory-related regions within the MTL, the memory-based RSM shows significant correlations (FDR corrected *α*** = **0.010) with several regions in the MTL: the left hippocampus (*r*(14) = 0.039, *p* = 0.0005), the right hippocampus (*r*(14) = 0.054, *p* = 0.0009), the left PHC (*r*(14) = 0.015, *p* = 0.010), the right PHC (*r*(14) = 0.016, *p* = 0.005), and with marginal significance (failing FDR correction) in the right PRC (*r*(14) = 0.008, *p* = 0.044) and the right amygdala (*r*(14) = 0.010, *p* = 0.026). The memorability-based RSM shows no significant correlations with any of the regions in the MTL (all *p* > 0.10). However, when RSAs are conducted only on trials split by memory performance, then we see these regions do contain memorability-related information for certain trial types. Several regions of the MTL do show a significant correlation (FDR corrected *α* = 0.039) with the memorability RSM for hit trials, specifically the right PRC (*r*(14) = 0.009, *p* = 0.023), the left PRC (*r*(14) = 0.013, *p* = 0.011), the left amygdala (*r*(14) = 0.015, *p* = 0.003), the left ERC (*r*(14) = 0.013, *p* = 0.006), the right ERC (*r*(14) = 0.012, *p* = 0.005), the left hippocampus (*r*(14) = 0.27, *p* = 0.015), the right hippocampus (*r*(14) = 0.025, *p* = 0.039), and the left PHC (*r*(14) = 0.024, *p* = 0.001). No significant effects were found for correct rejection trials; however, no significant difference was found between hit and correct rejection trials (*p* > 0.05). These results indicate that memorability is processed at the perceptual level regardless of image history or memory type. It is less clear what patterns in the MTL indicate about memorability, however these regions do appear to contain information about the memorability of a stimulus when successfully retrieving an old memory.

### Predicting Memorability Score from BOLD Activity Patterns

**Figure 6:**
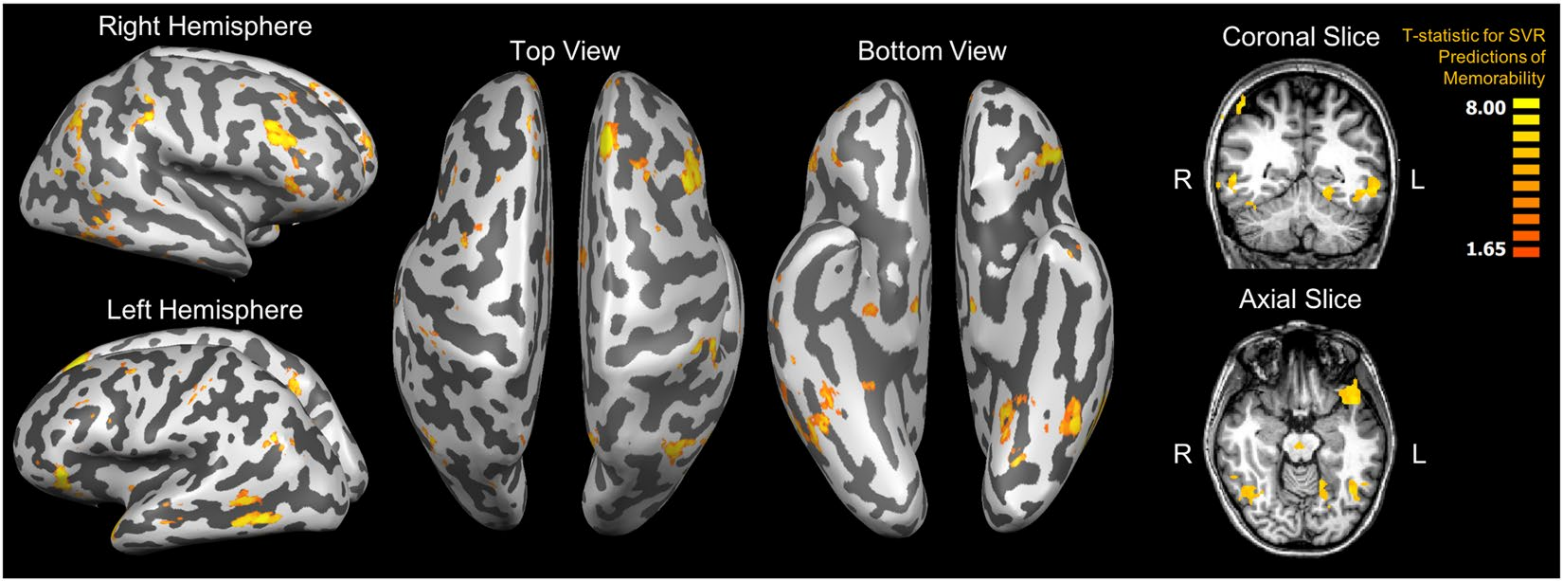
The top 1000 voxels whose local activity patterns showed significant correlations between a support vector regression’s predictions of items’ memorability and actual memorability scores. The lowest cluster-threshold corrected significance level that all 1000 voxels pass is *p* < 0.005.

A searchlight analysis of support vector regressions (80% training/20% testing split, 100 iterations) predicting memorability score from local multi-voxel activity patterns finds several similar regions significantly predictive of memorability score (Fig. 6), particularly two overlapping regions along the ventral visual stream and temporal lobe. ROI support vector regression analyses in the MTL regions find significant decoding accuracy bilaterally in the PHC (left: *r*(14) = 0.04, *p* = 0.034; right: *r*(14) = 0.02, *p* = 0.039) and the left hippocampus (*r*(14) = 0.06, *p* = 0.011), including its head (*r*(14) = 0.05, *p* = 0.008), body (*r*(14) = 0.06, *p* = 0.004), and tail (*r*(14) = 0.03, *p* = 0.026). No significant decoding accuracy was found in the PRC, ERC, amygdala, or right hippocampus (all *p* > 0.15). Frontal and parietal regions also appear here, similar to the RSA map for memory behavior, possibly because memorability score tracks with memory confidence (see Behavioral Results), and so regions sensitive to a subject’s memory strength may also be sensitive to a continuous metric of memorability. Importantly, previous decoding work has only focused on binary classifications of memory (i.e., remembered versus forgotten, memorable versus forgettable). However, these results indicate that the representation of memorability in the brain may be continuous rather than binary.

## Discussion

These results are the first to examine the interaction of item-centric memorability and observer-centric memory at the stage of memory retrieval. The two phenomena show separate areas of sensitivity, where sensitivity to stimulus memorability is observed in more ventral regions and encompasses the MTL, while sensitivity to memory retrieval outcomes is more apparent in frontal and parietal regions. In particular, the ventral visual stream holds a representational geometry where distributed neural ensembles respond more similarly for images of higher memorability, and less similarly for more forgettable images. This sensitivity to memorability for faces exists even in non-face selective regions (e.g., the PPA and LOC). Additionally, these results show that memorability is coded as a continuous metric within the brain, as memorability score can be predicted by several regions through support vector regressions.

These results mirror a dissociation recently found with memorability and memory *encoding* during perception of an image, where ventral perceptual and medial temporal lobe regions show sensitivity to stimulus-based memorability, while fronto-parietal regions show sensitivity to a participant’s successful encoding of an image^22^. While this previous study looked at memorability during an incidental encoding task (participants did a perceptual categorization task with no knowledge their memory would later be tested), in the current study, participants were performing an explicit retrieval task. The current results also show similar memorability processing in ventral perceptual regions regardless of the image’s memory history - whether it is an old image that is successfully remembered or a new image successfully identified as novel. Given both the current results and these previous results, the processing of the memorability of visual input may be a continuous and automatic process, that occurs during both encoding and retrieval, and during recollection/recognition and novelty detection. Memorability processing has been shown to temporally occur between early perception and the encoding of a stimulus^31^. Further, the current and past results show no sensitivity to memorability in early visual areas, reinforcing that the memorability of an image is not predicted merely by low level visual processing. The brain’s sensitivity to memorability may thus reflect a late perceptual process that determines what stimuli should be ultimately encoded into memory. Such a process may be supported in a feedforward manner by the ventral visual stream regions in the current study (regions along the inferotemporal cortex, overlapping with the FFA, PPA, and LOC); these regions may be identifying the memorability of images through a set of high-level perceptual features. Or, regions within the medial temporal lobe (PRC, PHC, hippocampus) could have an initial sensitivity to memorability, then causing a feedback-driven attentional signal, resulting in heightened response in these late perceptual regions (possibly explaining why scene-selective regions like the PPA show a sensitivity to memorable face images in the current study, due to a generally heightened response for memorable stimuli). Regardless of the region from which memorability sensitivity originates, the current results show a specific memorability-centric representational geometry in these sets of regions, where memorable stimuli are more neurally similar to one another, while more forgettable stimuli are more neurally dissimilar. These regions may support a statistical representation of our sensory experience that can distinguish certain stimuli as being more distinctive or memorable than others, and thus important to encode for later.

While the current study gives strong evidence for stimulus memorability as an important influence on neural patterns during a memory retrieval task, several new questions open up about the representation of memorability in the brain. While memorability has been shown to be an intrinsic property to a stimulus^1,13–21^ that is processed early during perception in the brain^19,31^, what are the perceptual features of an image that make it memorable? Memorability has been shown to be inexplicable by several other attributes such as attractiveness and subjective distinctiveness for faces^1^ and aesthetics and object content for scenes^16^. Viewing memorable and forgettable face and scene images controlled for several perceptual and other mid-level attributes (e.g., color, spatial frequency, gender, race, age, attractiveness, emotion, confidence, object content, scene category) during memory encoding still results in similar patterns in the brain as the current results^22^. This means that, for the time being, memorability-based brain effects cannot be explained by straightforward low-level visual and perceptual differences (e.g., differential symmetry based on attractiveness, differences in brightness between images). Computational models are beginning to gain moderate success at quantifying memorability from images^15^, and additional work will be necessary to fully understand the perceptual features that cause an image to be memorable. Faces, specifically, could serve as the perfect testbed as their features are often more physically quantifiable and manipulable in comparison to more unconstrained image categories such as scenes. For example, memorability could be tested in a data-driven manner for a large-scale set of face images that vary along all of the above-mentioned perceptual features.

Further work is also required to probe the precise statistical representations of these regions as they relate to the memorability of a stimulus, and the current RSAs could be expanded through varying other feature dimensions of the stimuli and examining the variance in memorability they can explain, or by looking at other distance metrics in the representational geometry. Similarly, while the current study finds generally similar effects of memorability for both hit and correct rejection trials, future experiments with higher trial counts for incorrectly performed trials (e.g., false alarms) could facilitate the investigation of how false recognition is influenced by stimulus memorability (e.g., what might be happening in the brain when certain novel stimuli consistently feel familiar?). Also, while the current study showed memorability sensitivity during correct rejection trials, might there be differential sensitivity for those novel stimuli that are correctly rejected but then successfully encoded versus those that are not (as could be assessed by a subsequent recognition test, like in^32^)? In other words, it is conceivable that the memorability effects we observed during retrieval may at least in part be a reflection of continuous encoding-related processes. Finally, as the current work expands our framework of memorability beyond a binary metric (i.e., memorable versus forgettable) to a continuous one (i.e., an image is 64% memorable), how might this continuous representation of memorability relate to a more fine-grained continuous metric of observer memory (e.g., a 20-point scale of memory strength as in^33^)?

In addition to furthering our understanding of the neural interaction between the memorability of a stimulus and the ultimate memory fate of that stimulus for an individual subject, these results also showcase how novel insights can be gained by reanalyzing existing fMRI datasets using new technologies and frameworks. While this dataset originally was used to examine the decoding of subject-specific memory performance from BOLD data^23^, the current experiment leverages the power of online crowd-sourced studies to collect large amounts of data about the stimuli themselves, in order to analyze item-level effects within the same neural data. Until now, most neuroimaging studies treat all exemplars of a stimulus category (e.g., faces, scenes, objects, words) as an equally good member. However, with recent strides in Big Data collection and analysis, researchers have begun to uncover subtle item-driven differences that may account for effects beyond just the stimulus condition. In the era of increased data sharing, so long as researchers share their full stimulus sets along with the fMRI data, many interesting and previously unforeseen explorations of their data may be possible.

## Methods

### fMRI experiment and data analysis

Please refer to^23^ for specific details on the data used in this experiment. Generally, 16 participants (10 female) were recruited for a face memory test where they studied 200 novel face images (4 s each) outside of the scanner, and then were tested for recognition with an event-related design inside the scanner approximately 1 h later. In the recognition test, the 200 original images were intermixed with an additional 200 novel foil images. Each image was shown for 2 s and participants noted their memory with five different options: (1) recollected the specific face, (2) high confidence for having seen the face, (3) low confidence for having seen the face, (4) low confidence for it being novel, and (5) high confidence for it being novel. There was a 8 s fixation interval between each image. Written informed consent was obtained for all participants under a protocol approved by the Institutional Review Board (IRB) at Stanford University, and all methods for this study were performed in accordance with the regulations set by the Stanford University IRB.

The experiment was conducted on a 3 T GE Signa MRI system (TR = 2 s, TE = 30 ms, flip angle = 75, FoV = 22 cm, in-plane resolution = 3.44 mm × 3.44 mm × 4.00 mm). Data were preprocessed with SPM5 (www.fil.ion.ucl.ac.uk/spm), including motion correction with a two-pass, six-parameter, rigid-body realignment procedure. Each time series was also high-pass filtered, detrended, and z-scored. Voxel data were taken as the average of the time points at the peak of the hemodynamic response (the 3rd and 4th TRs relative to face onset, representing BOLD effects measured 4–8 s post-stimulus). No spatial smoothing was applied. For visualization purposes, results were transformed to the Talairach atlas space, and are displayed on a model inflated brain. Figures were produced using BrainVoyager QX 2.8 (http://www.brainvoyager.com/)^34^.

### Region of interest (ROI) specifications

In the current experiment, key ROIs in the MTL and ventral visual stream were identified in each participant: the hippocampus (including hippocampal head, body, and tail), amygdala, ERC, PRC, PHC, FFA, PPA, LOC, and EVC. These regions were identified using a probabilistic map of voxels shared by at least 25% participants (a common threshold used in other work: e.g.^30^,) based on ROIs determined from previous studies, which included 40 participants for MTL regions and 70 participants for ventral visual stream regions^22^. The original MTL ROIs were manually segmented using MTL anatomical landmarks^35,36^, while ventral visual stream ROIs were determined based on independent functional localizers containing face, scene, object, and scrambled images. The probabilistically determined ROIs were then transformed into each participant’s native subject space for analyses.

### Memorability test and consistency analysis

A face memory test was conducted on AMT, an online experiment crowd-sourcing platform, as in^1^. A total of 941 workers (404 female; age: *M* = 34.94, *SD* = 11.14, *range*: 18–76) participated in the experiment, first providing informed consent following the protocol guidelines approved by the IRB at the Massachusetts Institute of Technology. The methodology for this memory test was performed in accordance with these IRB guidelines. In the test, participants saw a continuous stream of face images and pressed a key whenever they saw an image they had seen previously during the experiment. Each image was shown for 1.0 s with a 1.4 s interstimulus interval. 80 of the images (20%) shown were “target” images that repeated during the experiment 91–109 images after their first presentation. The remaining 320 images (80%) were “filler” images, used as spacing between target images, and sometimes repeating soon after their first presentation (1–7 images apart) to ensure that participants were paying attention to the task. There was no difference between target and filler images from the perspective of the participant, as they were continuously identifying repeats (unaware of target or filler status of each image), but memorability scores were only determined for the targets, because of the longer delay between their repetitions. In order to ensure participants were attentive to the task, if participants made more than 50% false alarms or missed more than 50% image repeats for the filler images, then they were prevented from continuing with the task, and their data were not incorporated into our analyses. 201 participants failed these criteria, and so ultimately the data from 740 (323 female; age: *M* = 34.50, *SD* = 10.97, range: 18–76) participants were used in the analyses. Targets and fillers were counterbalanced across participants, so ultimately approximately 50 participants made a target repeat response on each of the 400 images.

Memorability consistency was assessed through split-half ranking correlations^13^. Essentially, images were ranked by memorability score (*HR*, *FA*, *Pr*, or *d-prime*) determined from one random half of the participants and these scores were correlated with those of the same image ordering for the second half of participants, using Spearman’s rank correlation tests. These split halves were repeated over 10,000 iterations, and then averaged to get a final consistency score. This metric indicates to what degree memorability scores are stable in an image, across different groups of observers. Chance in this analysis represents the average consistency if the image ordering was randomly permuted in the second half of participants before correlating, and p-values represent the results of a permutation test.

### Representational similarity analyses (RSAs)

RSAs allow the testing of specific hypotheses of how information may be represented in the brain, as well as directly comparing different hypotheses^37^. In the current experiment, hypotheses were defined based on stimulus memorability as well as subject memory and then contrasted. Hypotheses are formed through a representational similarity matrix (RSM), indicating hypothesized degree of similarity (or dissimilarity) between every stimulus pair. The hypotheses were selected based on winning hypotheses found in^22^ (the only hypothetical models significantly correlated with voxel patterns in perceptual and memory-related ROIs), but are now expanded to use memorability as a continuous value rather than as a binary category type. This previous work used images at the extreme ends of memorability (i.e., binary categories of highly memorable and highly forgettable images), and found that regions across the brain showed significant correlations to two possible hypothesized models^22^: (1) the more memorable an image is, the more neurally similar it is, and (2) the more remembered an image is for an individual participant, the more neurally similar it is (i.e., similar activity patterns during encoding are predictive of subsequent memory). These models are akin to other work showing there are regions in the brain that show more similar neural patterns for images that are ultimately remembered^38–42^. In the current study, using memorability as a continuous metric allows us to examine more fine-grained hypotheses about the representation of memorability in the brain, not previously possible in prior memory work.

To construct an RSM based on image memorability, pairwise similarity was taken as the mean *Pr* of every possible pair of two stimuli across all response types. With this model, images would be hypothesized to be most neurally similar to the most memorable image; i.e., two memorable images would be neurally similar, and forgettable images would be more neurally similar to memorable images than other forgettable images (Fig. 3). This would predict a memorability-centric geometry, where items that are memorable take on a more stereotyped neural pattern, whereas items that are forgettable have no specific pattern. This geometry has been shown previously for encoding of items that are subsequently remembered versus forgotten by participants^38–42^. A subject memory-based hypothesized RSM was also constructed for all previously studied stimuli, except using binary values based on individual subject memory performance (i.e., remembered = 1, forgotten = 0) rather than stimulus memorability, and then taking the mean memory performance for each image pair. Thus, pairs of images where both were remembered were coded with a 1, pairs of images where one was remembered and one was forgotten was coded with a 0.5, and pairs of images where both were forgotten was coded with a 0. Ultimately, each participant ends up with their own individualized memory-based hypothesis RSM, while the memorability-based hypothesis RSM is stimulus-centric and thus the same across all participants.

Once hypothesized memorability-based and memory-based RSMs are defined, they are then compared to the real data-based RSMs (Fig. 3). 7-voxel diameter spherical searchlights were moved through the brain and data-based RSMs were formed through Pearson’s correlations between the voxel values (BOLD signal change averaged at the 3rd and 4th TRs relative to stimulus onset) within that sphere for every image pair. Hypothesized RSMs and data-based RSMs were then compared using Spearman’s rank correlations at each spherical searchlight in the brain, resulting in a whole-brain correlation map for each participant of the degree to which actual BOLD data relates to that hypothesis. The correlation maps were then transformed with Fischer’s z-transformations to allow parametric statistics to be performed on them. Group whole-brain maps were created by normalizing all participants to Talairach space and then performing *t*-tests of the whole-brain maps of the sixteen participants versus a null hypothesis of no correlation (*r* = 0). The resulting maps show where in the brain each hypothesis is significantly correlated with the BOLD data, across participants. As there were large differences between the effects being compared (for example, subject-based memory behavior has more widespread effects as it is tied to the task, motor response, and participants’ decisions, while stimulus memorability was not a task-relevant variable per se, and was only parameterized post-hoc), we show the comparison of any two maps by showing the top 1000 significant voxels for each map. ROI analyses were also conducted using the same methods, but by extracting data-based RSMs from the voxels within probabilistically defined ROIs rather than spherical searchlights.

The memorability-based RSA was done including all 400 stimuli regardless of image history (old/new) and participant response (confidence). In addition, we conducted separate memorability-based RSAs for specific memory response types (i.e., hit trials or correct rejection trials). For hit trials, we took all old trials with a correct old response (recollection, high confidence old, low confidence old), and for correct rejection trials, we took new trials with a correct new response (high confidence new, low confidence new). Confidence levels were not separated because of the loss in power from the decrease in trial counts (i.e., on average there were 135 hit trials, and of those, 36.4 recollection responses, 43.2 high confidence, and 55.3 low confidence). We downsampled the data so that the number of trials was the same across trial types. To do this, for each participant, we identified the minimum number of trials *m* between the two types (hit trials, correct rejection trials). For the trial type with more than *m* trials, we took a random subset of *m* trials and conducted the RSM with that subset of trials. This way, any effects we find are not due to there being more trials of one memory response type than the other. Participants generally had high numbers of both trial types, with on average 135 hit trials (*SD* = 22.9, *min* = 93, *max* = 168), and 138 correct rejection trials (*SD* = 25.3, *min* = 89, *max* = 177).

### Support vector regression (SVR) analyses

SVRs were used to predict memorability score *Pr* from BOLD activity. SVRs allow us to determine the degree to which a representation of memorability along a continuous scale exists in the brain. *Pr*s were first logit-transformed to allow for linear statistics. SVRs were conducted with 100 iterations, using 80% of the trials for training and 20% of the trials for testing with no replacement. Accuracy was taken as the mean Pearson’s correlation coefficient between predicted *Pr*s and actual *Pr*s over the 100 iterations. For the whole brain analyses, SVRs were conducted in a searchlight sphere of 7-voxel diameter moved throughout the brain. For the ROI analyses, SVRs were conducted in the probabilistically-defined ROIs.

### Data availability

No new datasets were generated during the current study, however tools for doing these analyses (i.e., measuring the memorability of stimuli from a prior study) are available on the corresponding author’s website.

## Electronic supplementary material

Supplementary Information
