# Supplementary material for: Dissociating neural markers of stimulus memorability and subjective recognition during episodic retrieval
Source: Sci Rep. 2018 Jun 6;8:8679. doi: 10.1038/s41598-018-26467-5 (PMC5989217; doi:10.1038/s41598-018-26467-5)
Supplement: Supplementary file 1 — Supplementary Information [file 41598_2018_26467_MOESM1_ESM.docx]

**Supplementary Information:**

**Dissociating neural markers of stimulus memorability and subjective recognition during episodic retrieval**

Wilma A. Bainbridge* (1) and Jesse Rissman (2,3)

1 – Department of Brain and Cognitive Sciences, Massachusetts Institute of Technology, Cambridge MA

2 – Department of Psychology, University of California, Los Angeles, Los Angeles CA

3 – Department of Psychiatry and Biobehavioral Sciences, University of California, Los Angeles, Los Angeles CA

Supplementary Information


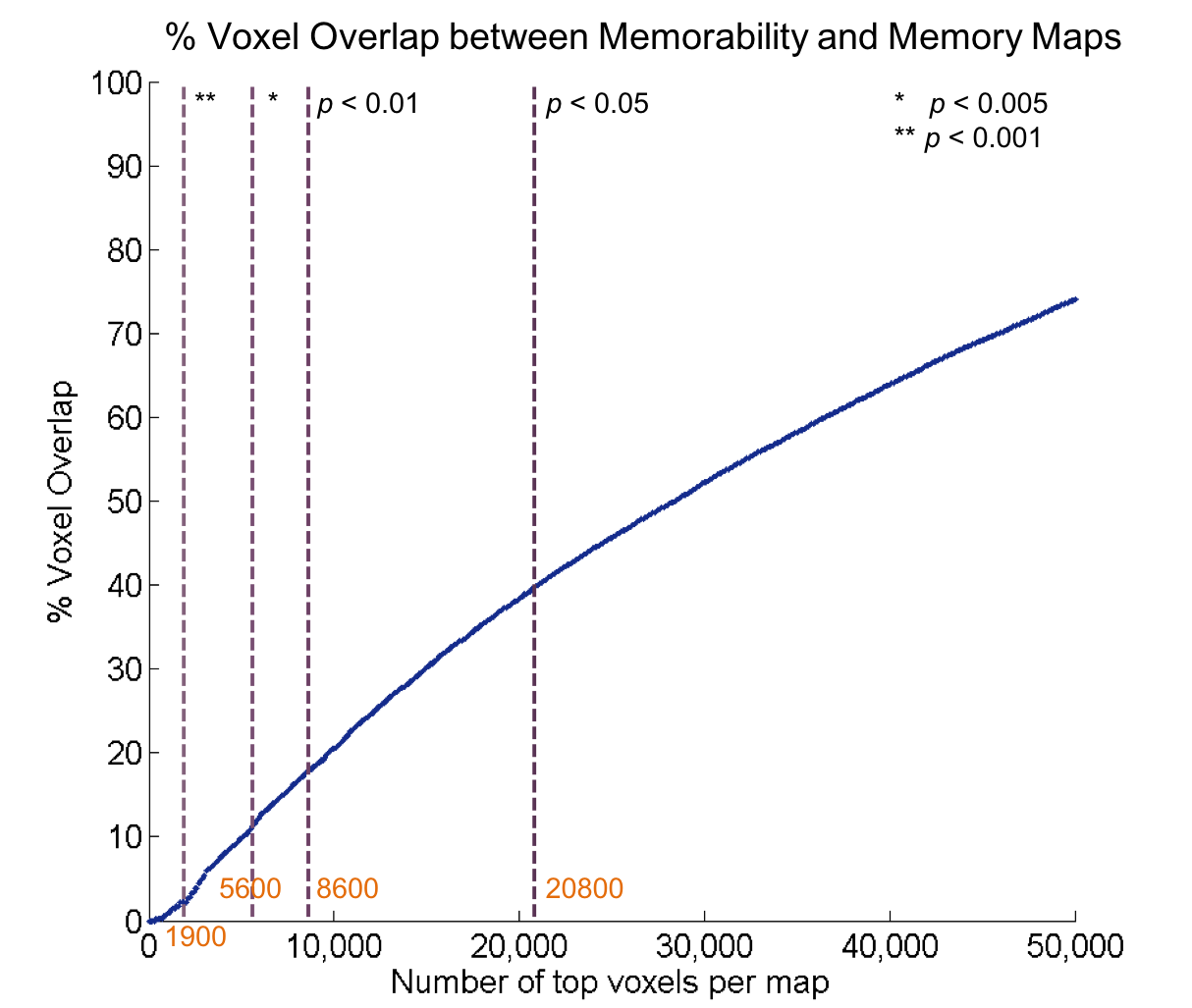


Figure S1. Chart of the % voxel overlap between memorability and subject-based memory t-maps maps across 16 participants (as in Figure 4) as a function of number of top-ranked voxels included in the maps (voxels were ranked from highest to lowest correlation with the memorability or memory model RSM). Dashed vertical lines indicate the points at which voxels in both maps pass a specified significance threshold (post cluster-threshold correction), with orange labels indicating the number of voxels. For example, the top 8,600 voxels for both maps all pass a threshold of *p* < 0.01, and show less than 20% overlap between the memorability map and memory map. Beyond 20,800 voxels, the maps no longer contain solely significant data. The amount of overlap is relatively low at more stringent significance levels (less than 10% at *p* < 0.005), and gradually increases as more voxels are added. The original brain maps contained 153,594 voxels total.


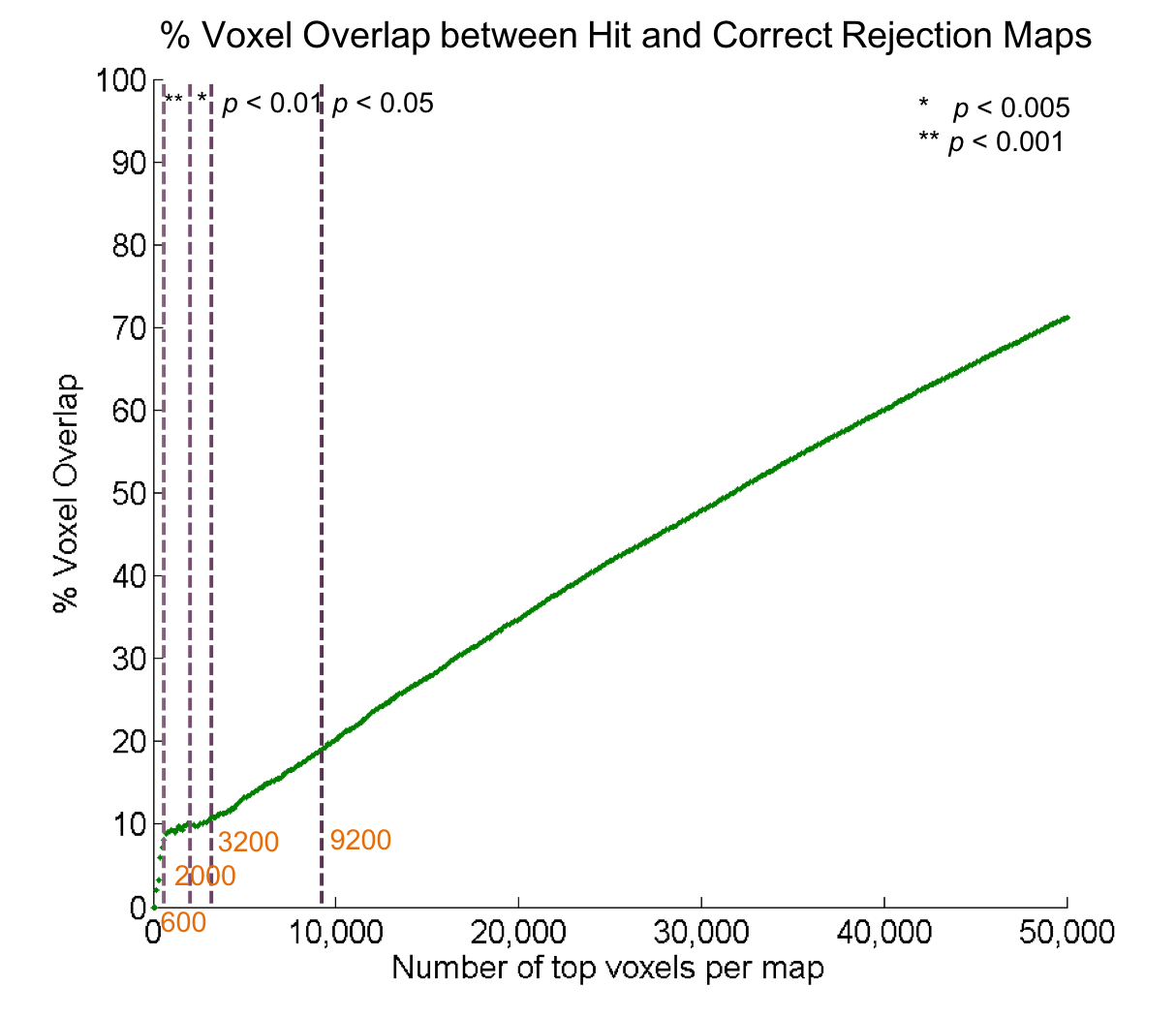


Figure S2. Chart of the % voxel overlap between the memorability maps for hit trials and correct rejection trials across 16 participants as a function of number of top-ranked voxels included in the maps (voxels were ranked from highest to lowest correlation with the memorability model RSM using hit trials or correct rejection trials). This chart follows the same layout as Figure S1. The amount of overlap quickly rises for the first several hundred voxels, and then gradually increases after approximately 600 voxels, indicating that there is a relatively high degree of overlap for those voxels showing the very strongest effects in each map.
